# From Centralized to Distributed Entropy: Long-Term Resilience and Structural Evolution of Regional Innovation Networks in the Yangtze River Delta

**DOI:** 10.3390/e28070826

**Published:** 2026-07-20

**Authors:** Ju Yang, Fenglei Zhou, Zhongying Hu, Lintao Zha, Hui Yang

**Affiliations:** 1 Department of Economics and Management, West Anhui University, Lu’an 237012, China; 10000053@wxc.edu.cn (J.Y.); 10000086@wxc.edu.cn (L.Z.); 10000060@wxc.edu.cn (H.Y.); 2 Department of Economics and Management, Huazhong Agricultural University, Wuhan 430070, China; 3 College of Science and Technology, Nanchang Hangkong University, Gongqingcheng 332020, China

**Keywords:** innovation networks, network resilience, polycentric development, weighted-degree entropy, patent cooperation, Yangtze River Delta

## Abstract

Understanding how regional innovation networks maintain functionality under disruption is critical for regional economic sustainability. This study investigates the structural evolution and topological resilience of inter-city patent cooperation networks in China‘s Yangtze River Delta (YRD) from 2005 to 2024. We construct weighted networks of 41 cities and simulate targeted attack scenarios to quantify network resilience as the area under the robustness curve. Using degree distribution entropy to quantify the spatial distribution of innovation activity, we demonstrate that the system’s innovation activity redistributes from a Shanghai-dominated, concentrated, single-core configuration to a more distributed, polycentric architecture. The resilience index increases from 0.0836 in 2005 to 0.5000 in 2015, and full connectivity is achieved by 2024. Notably, removing the top-ranked node in 2010 reduces the largest connected component by 5.3%, whereas removing four core nodes simultaneously in 2015 produces a 9.8% reduction, indicating that the redistribution of innovation activity is associated with systemic robustness. Correlation analysis further reveals strong associations between resilience and network density, weighted-degree entropy, and short path lengths, reflecting a hub-dominated topology that evolved from a single hub (Shanghai) to multiple co-existing hubs (Shanghai, Nanjing, Hangzhou, Hefei). These findings provide empirical evidence consistent with the view that polycentric configurations are associated with innovation ecosystem resilience and offer actionable insights for regional sustainability policies.

## 1. Introduction

The Yangtze River Delta (YRD), encompassing Shanghai Municipality and the provinces of Jiangsu, Zhejiang, and Anhui, is one of China’s most economically dynamic and innovation-intensive regions. Since its elevation to a national development strategy in 2018, the YRD has prioritized the construction of a science and technology innovation community to enhance cross-regional collaborative capacity through shared innovation resources [1]. Fostering new quality productive forces requires strengthening the coordination of innovation resources and leveraging the synergistic effects among cities [2]. However, technological innovation has become increasingly complex, characterized by growing interdisciplinarity and cross-sectoral integration, making it difficult for any single city to achieve major breakthroughs independently [3]. Innovation activities are no longer confined to isolated urban units but are embedded in complex inter-city networks [4]. As economic activities shift from a “space of places” to a “space of flows” [2], cities have transformed from agglomeration points into network nodes, embedding themselves into local and global innovation systems [5]. Understanding the structural characteristics and evolutionary patterns of collaborative innovation networks within urban agglomerations is therefore critical for optimizing regional innovation resource allocation. In particular, network centrality has been shown to significantly enhance urban green total factor productivity, underscoring the relevance of network analysis for sustainable development.

A growing body of literature has significantly advanced our understanding of these networks. Core–periphery structures have been documented in the Beijing–Tianjin–Hebei, YRD, and Guangdong–Hong Kong–Macao Greater Bay Areas, while a polycentric spatial pattern has been identified in the YRD, with Shanghai, Suzhou, Nanjing, Hangzhou, and Hefei as core nodes [6,7]. Longitudinal studies reveal a sustained upward trend in the regional collaborative innovation index and a three-stage development trajectory—low progressive growth, steady growth, and rapid escalation [4,8]. Complementing these findings, network-oriented research has identified cohesive subgroups and hub-dominated structures, with core cities acting as “technology gatekeepers” and networks shifting from single-center to multi-center configurations [9,10,11,12].

More recently, attention has turned to the mechanisms underlying the formation and evolution of these networks, as well as their innovation effects. Drawing on multidimensional proximity theory, studies have found that geographical, institutional, and organizational proximity significantly promote inter-city innovation cooperation [13]. Inter-city collaboration is shaped by both endogenous and exogenous factors [9]. In terms of effects, disruptive technology innovation networks enhance regional innovation efficiency [3], and the robustness of urban knowledge networks correlates positively with innovation performance [14]. Furthermore, the national strategy of YRD integration has generated significant “innovation cooperation creation effects” [1,15,16]. Recent comparative studies have further revealed that knowledge and technology innovation networks exhibit distinct resilience patterns under different attack scenarios, highlighting the importance of network architecture for sustainable urban innovation.

Despite these important contributions, three critical gaps remain.

First, existing resilience studies have not quantitatively linked long-term network evolution to measured improvements in resilience. Several recent studies have assessed the resilience of the YRD innovation network using attack simulations [7,8]; however, they have focused on static network properties at individual time points. How the long-term evolutionary process—specifically, the transition from a single-core to a polycentric configuration—quantitatively enhances resilience has not been empirically traced over a two-decade horizon. This gap limits our understanding of the dynamic relationship between structural change and systemic robustness.

Second, the role of network resilience as a mediator between structural evolution and innovation outcomes remains underexplored. While network evolution [4,9] and innovation performance [3,14] have been studied separately, few studies have examined whether and how improvements in resilience—driven by structural reconfiguration—translate into regional innovation capacity. This gap obscures the functional significance of network resilience for sustainable regional development.

Third, the impact of integration policies on network architecture, rather than merely on collaboration volume, has not been empirically tracked over time. Although the positive effects of the YRD integration policy on innovation cooperation have been documented [1,17], how such policies reshape network topology—particularly the shift toward polycentricity—and thereby influence systemic robustness remains unclear. Addressing this gap is essential for designing evidence-based regional innovation policies that enhance long-term adaptive capacity.

To address these gaps, this study investigates the structural evolution and resilience of collaborative innovation networks in the YRD from 2005 to 2024. Using inter-city patent cooperation data, we employ social network analysis to characterize network structural features and simulate targeted node removal scenarios to quantify network resilience. Our analysis reveals a four-stage evolutionary pathway: node polarization (2005–2010), cluster emergence (2010–2015), system restructuring (2015–2020), and full integration (2021–2024). The pathway culminates in a fully connected polycentric configuration by 2024. More importantly, we provide direct empirical evidence that the transition to a polycentric architecture substantially enhances the network’s ability to withstand core node failures. A counter-intuitive finding also emerges: full connectivity achieved in 2024 did not restore the resilience peak of 2015, challenging the assumption that more connections always yield greater robustness.

This study contributes to the literature in three ways. First, it provides empirical evidence that the transition toward a polycentric architecture substantially enhances network resilience, and reveals that full connectivity does not guarantee maximal resilience. Second, it constructs an integrated analytical framework linking network evolution, node functionality, and systemic resilience, offering a replicable methodology for assessing the long-term viability of innovation ecosystems. Third, it identifies density, average degree, degree entropy, and average path length as key topological features associated with resilience in a hub-dominated innovation network that has evolved toward polycentricity.

The remainder of this paper is organized as follows. Section 2 reviews the relevant literature and identifies research gaps. Section 3 describes the data sources and methodology. Section 4 presents the empirical results and discussion. Section 5 concludes with policy implications and directions for future research.

## 2. Materials and Methods

### 2.1. Study Area

The study area is the Yangtze River Delta (YRD) region as officially defined by China’s National Development and Reform Commission. The YRD comprises 41 prefecture-level and above cities across Shanghai Municipality and the provinces of Jiangsu, Zhejiang, and Anhui [7]. Covering approximately 358,000 km^2^, this region accounts for about one-quarter of China’s gross domestic product and one-third of its patent applications, establishing it as one of the most economically dynamic and innovation-intensive areas in the country [10].

Three considerations justify the selection of the YRD as the study area: (a) since its elevation to a national strategy in 2018, the YRD has been at the forefront of China’s regional integration initiatives [1]; (b) the region possesses a well-developed innovation ecosystem, characterized by dense inter-city economic and technological linkages [11]; and (c) the availability of comprehensive patent data, combined with the region’s status as a major hub for technological innovation, makes it an ideal case for studying collaborative innovation networks [9].

Table 1 lists the 41 cities included in this study. To avoid ambiguity, cities with identical names are distinguished by province in parentheses. The study period spans from 2005 to 2024. To capture long-term evolutionary dynamics, four time points with five-year intervals (2005, 2010, 2015, 2020) are selected, aligning with China’s national five-year planning cycles [11]. In addition, annual data from 2021 to 2024 are included to analyze the most recent dynamics.

### 2.2. Data

Patent cooperation data are used to construct inter-city collaborative innovation networks. Patents are widely recognized indicators of innovation output, and joint applications effectively capture knowledge-sharing relationships among different entities [11,12]. Invention patents, which embody higher technological content, are particularly suitable for measuring collaborative innovation [3].

This study integrates data from two sources. For the period 2005–2020, pre-processed data were obtained from the National Earth System Science Data Center (http://www.geodata.cn (accessed on 24 March 2026)), a nationally recognized scientific data infrastructure. This dataset provides the total number of cooperative invention patents between each unordered pair of the 41 YRD cities at the four designated time points, as listed in Table 1. Since this dataset is available only up to 2020, data for 2021–2024 were directly retrieved from the China National Intellectual Property Administration (CNIPA) Patent Search and Analysis System (https://pss-system.cponline.cnipa.gov.cn/) (accessed on 26 March 2026).

To ensure cross-period comparability between the two data sources, we conducted a consistency check using the overlapping year 2020. We retrieved raw data from CNIPA for 2020 using the same search criteria as for the 2021–2024 period and compared the resulting city-pair cooperation counts with those from the pre-processed dataset. The two sources yielded a Pearson correlation of 0.9363 (*p* < 0.001) and a Spearman correlation of 0.9906 (*p* < 0.001) at the city-pair level. The total number of cooperative patents was 8400 (pre-processed) and 7408 (CNIPA), representing a difference of 11.81%. Both datasets contained 314 edges, with a non-zero edge matching rate of 100%. The mean absolute percentage error (MAPE) was 4.49%, and 93.6% of city-pairs had an absolute error of 5 patents or fewer. These results confirm that the two data sources are highly consistent, supporting the validity of cross-period comparisons. The CNIPA retrieval protocol for 2021–2024 followed these criteria: (1) application type: invention patents only; (2) time window: application year (not publication year); (3) applicant identification: all applicants on each patent record, with city identification based on the applicant’s registered address; (4) cooperation definition: a patent is counted as a collaboration if it lists at least two applicants from different cities; and (5) deduplication: multiple applicants from the same city on a single patent are counted once for that city-pair, and multiple patents between the same city-pair are aggregated.

### 2.3. Network Construction and Measurements

#### 2.3.1. Network Construction

For each of the eight time points, we constructed an undirected weighted network in which cities represent nodes and inter-city patent cooperation counts represent edges. For each patent record, we identified the cities of all applicants and recorded the cooperation relationship. We then constructed a 41 × 41 weighted adjacency matrix for each time point. Diagonal elements were set to zero to exclude intra-city cooperation, and each off-diagonal element $w_{ij}$ denotes the total number of cooperative patents between city *i* and city *j*. Following established practice [13], we retained all connections with at least one cooperative patent, consistent with previous studies on Chinese urban innovation networks [6]. The matrices were then saved as CSV files and loaded into Python (v3.14) using the NetworkX library (v3.6.1) for subsequent network analysis.

#### 2.3.2. Network-Level Measurements

The following network indicators were calculated using Python 3.14 with the NetworkX library (version 3.6.1).

Network density measures the proportion of actual ties to all possible ties in the network. It is calculated as:

(1) D=2m/[n(n−1)]

where m is the number of actual ties and n is the number of nodes. Higher density indicates more extensive collaboration.

Average clustering coefficient measures the degree to which a node’s neighbors are connected to each other. The local clustering coefficient for node i is:

(2) $$C_{i}=2E_{i}/[K_{i}(K_{i}-1)]$$

where $E_{i}$ is the number of edges among the neighbors of i and $K_{i}$ is the degree of i. The average clustering coefficient is the mean of $C_{i}$ over all nodes [18].

Average path length is the average number of steps along the shortest paths for all possible pairs of network nodes:

(3) $$L=\frac{1}{n(n-1)}\sum_{i\neq j}d_{\mathrm{ij}}$$

where $d_{\mathrm{ij}}$ is the shortest path distance between nodes i and j [19].

For weighted networks, we adopt the weighted clustering coefficient proposed by Barrat et al. (2004) [18]:

(4) $$C_{i}^{w}=\frac{1}{s_{i}(k_{i}-1)}\sum_{j,h}{\frac{\left(w_{ij}+w_{ih}\right)}{2}a}_{\mathrm{ij}}a_{ih}a_{jh}$$

where $s_{i}$ is the weighted degree of node *i*, $k_{i}$ is its unweighted degree, $w_{ij}$ is the weight of the edge between *i* and *j*, and $a_{ij}$ indicates the presence (1) or absence (0) of an edge. This metric accounts for both the number and intensity of connections among a node’s neighbors.

#### 2.3.3. Node-Level Centrality Measurements

Degree centrality captures the number of direct ties a node has. For node i:

(5) $$C_{D}(i)=\sum_{j}x_{\mathrm{ij}}$$

where $x_{\mathrm{ij}}=1$ if cities i and j are connected and 0 otherwise. This measure identifies cities with the most collaborative partners [20].

Core–periphery structure is identified by computing the coreness score for each node using the NetworkX function networkx.core_number() [21], which assigns higher values to nodes that are more densely connected to other core nodes. This distinguishes a cohesive core set of nodes from a peripheral set with fewer ties to the core.

#### 2.3.4. Structural Hole Measurements

Following Burt [22], we calculated three structural hole indicators using Python with the NetworkX library (version 3.6.1) to capture the extent to which a node occupies a bridging position.

Effective size is the number of non-redundant contacts a node has:

(6) $${\mathrm{ES}}_{i}=\sum_{j}(1-\sum_{q}p_{\mathrm{iq}}m_{\mathrm{iq}})$$

where $p_{\mathrm{iq}}$ is the proportion of node i’s relations invested in node q, and $m_{\mathrm{iq}}$ is the marginal strength of the connection between j and q. Higher effective size indicates greater access to non-redundant information.

Efficiency is the ratio of effective size to actual degree:

(7) $$E_{i}={\mathrm{ES}}_{i}/k_{i}$$

where $k_{i}$ is the degree of node i. Efficiency measures the information benefits per connection.

Constraint measures the degree to which a node is constrained by its connections:

(8) $$C_{i}={\sum_{j}\left(p_{\mathrm{ij}}+\sum_{q}p_{\mathrm{iq}}p_{\mathrm{qj}}\right)}^{2}$$

where $p_{\mathrm{ij}}$ is the proportion of node i’s relations invested in node *j*. Lower constraint indicates greater structural hole advantages.

### 2.4. Network Resilience Assessment

To assess network resilience against the failure of core nodes, i.e., the most critical disruption scenario for collaborative innovation networks, this study simulates targeted node removal scenarios [23]. This approach allows us to evaluate whether the evolving polycentric structure enhances robustness against deliberate disruptions to central hubs.

#### 2.4.1. Simulation Protocol

For each time point, we simulated targeted node removal: nodes were removed in descending order of degree centrality, representing a scenario in which central hubs are deliberately disrupted [23]. For each removal step, we removed nodes sequentially and calculated the size of the largest connected component after each removal step. This design focuses on the network’s capacity to withstand the loss of its most influential nodes, which is particularly relevant for understanding the resilience of innovation networks where core cities play critical roles in knowledge diffusion and collaboration.

#### 2.4.2. Resilience Metrics

Following established methods [24], we calculated the following resilience metrics.

Network robustness R(f) is the proportion of nodes remaining in the largest connected component after a given proportion of nodes has been removed, calculated as:

(9) $$R\left(f\right)=C_{\max}(f)/N$$

where $C_{\max}\left(f\right)$ is the size of the largest connected component after removing fraction f of nodes, and N is the total number of nodes.

Resilience index Ω is the area under the curve, calculated as:

(10) $$\Omega =\int_{0}^{1}R(f)\mathrm{df}$$

Higher values indicate greater resilience. This index provides a single summary measure of network robustness across the entire range of node removal, enabling direct comparison across different years and network configurations.

### 2.5. Correlation Analysis

To examine the relationship between network structural characteristics and resilience, we employed Pearson correlation analysis. The structural characteristics include network density, connectedness, average clustering coefficient, average path length, and degree entropy. The resilience index was derived from the targeted attack simulation described in Section 2.4. The Pearson correlation coefficient is calculated as:

(11) $$r=\frac{\sum_{i=1}^{n}\left(x_{i}-\bar{x}\right)(y_{i}-\bar{y})}{\sqrt{\sum_{i=1}^{n}{\left(x_{i}-\bar{x}\right)}^{2}}\sqrt{\sum_{i=1}^{n}{\left(y_{i}-\bar{y}\right)}^{2}}}$$

where $x_{i}$ represents a structural characteristic indicator for year i, $y_{i}$ represents the resilience index for year i, and $\overset{-}{x}$ and $\overset{-}{y}$ are the respective means.

### 2.6. Software and Implementation Details

All network analyses were implemented in Python 3.14 with NetworkX 3.6.1 and NumPy 2.4.4. Network indicators were computed using the following NetworkX functions: nx.density() (density), nx.average_clustering() (binary clustering coefficients), nx.average_shortest_path_length() (average path length), nx.connected_components() (connectivity), and nx.degree() (degree centrality). The evolutionary stage characteristics are summarized. Structural hole indicators were computed using nx.constraint() and nx.effective_size(). Targeted node removal simulations and resilience index *Ω* computation were performed using custom Python scripts. The weighted clustering coefficients were computed following the Barrat et al. (2004) [18] formula as defined in Equation (4). All code and data are publicly available (see Data Availability Statement).

## 3. Results

### 3.1. Overall Network Evolution

Table 2 presents the overall characteristics of the YRD patent cooperation network from 2005 to 2024. The results reveal a significant deepening of inter-city patent cooperation over the study period.

Network density increased from 0.052 in 2005 to 0.548 in 2020, then moderated to 0.182–0.220 during 2021–2024. Average weighted degree increased from 5.56 in 2005 to 673.41 in 2020, reflecting the growing volume of cooperative patents per city. The lower average weighted degree values in 2021–2024 (174–194) reflect a moderation after the peak in 2020, yet remain substantially higher than pre-2015 levels, indicating sustained high levels of inter-city collaboration.

The weighted average clustering coefficient remained low throughout the study period, ranging from 0.0095 to 0.0394, indicating that the network does not form dense local clusters. Meanwhile, the average path length declined from 2.186 in 2005 to 1.452 in 2020, then remained stable between 1.9 and 2.0 during 2021–2024. The combination of low clustering and short path lengths throughout the post-2010 period reflects a hub-dominated architecture: a few core cities (Shanghai, Nanjing, Hangzhou, Hefei) maintain direct connections to most other cities, enabling efficient global reach without forming dense local clusters.

Table 3 reports network connectivity and fragmentation. Fragmentation, which measures the proportion of unreachable node pairs, decreased dramatically from 68.3% in 2005 to 7.2% in 2015 and remained at that level through 2020. The trend continued after 2020: fragmentation dropped further to 4.9% during 2021–2023, and reached 0% in 2024, meaning that all 41 cities became fully connected in a single component.

### 3.2. Network Evolution Stages

**Figure 1:**
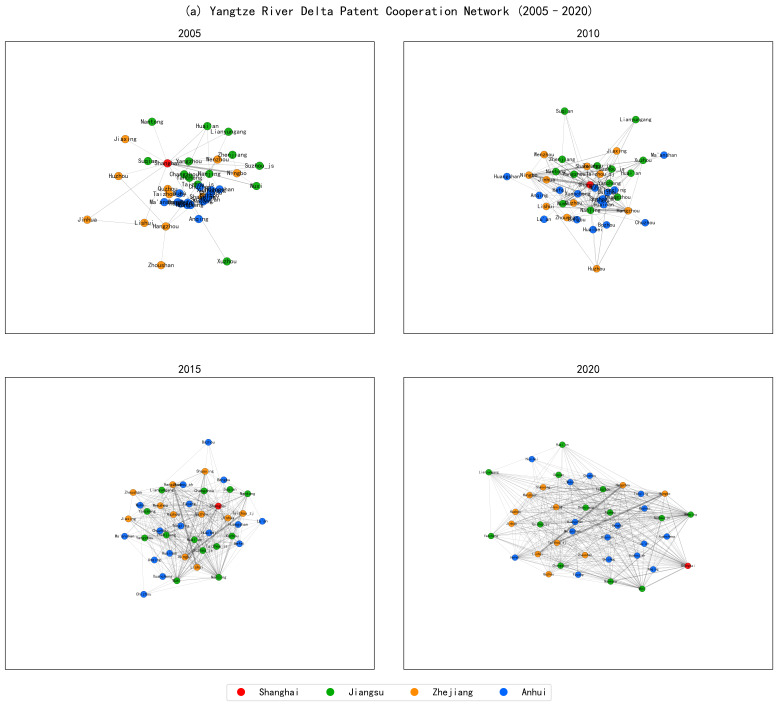

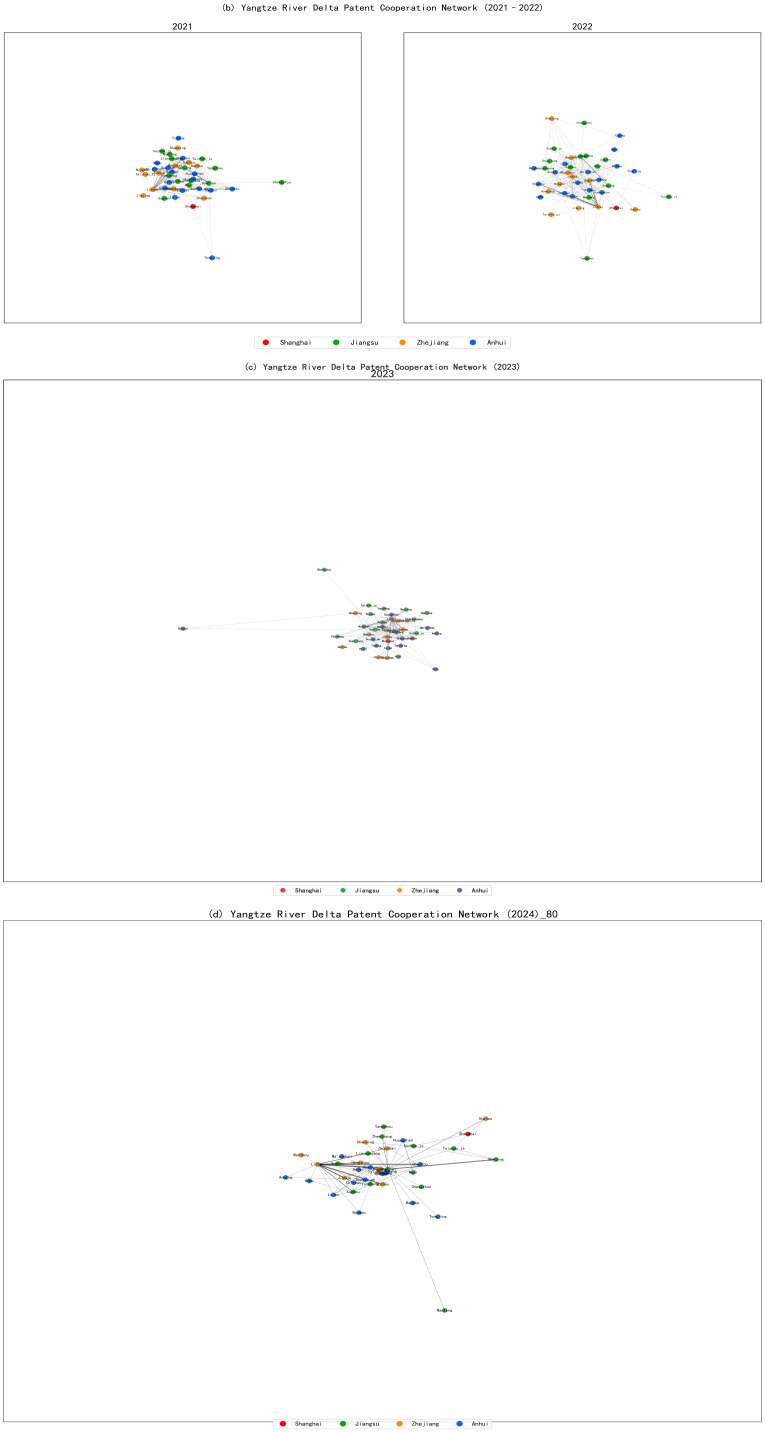
Structural evolution of the Yangtze River Delta patent cooperation network (2005–2024), presented in four panels to accommodate the substantial increase in network density over time. (**a**) 2005–2020 (2 × 2 layout); (**b**) 2021–2022 (1 × 2 layout); (**c**) 2023 (enlarged); (**d**) 2024 (enlarged, showing top 30 strongest ties out of 180+ total edges). In all panels, nodes represent cities; node colors indicate provincial affiliation (red: Shanghai; green: Jiangsu; orange: Zhejiang; blue: Anhui). Edge thickness is proportional to patent cooperation intensity. The layout employs the Kamada–Kawai spring embedding algorithm. The full 2024 network is provided in Supplementary Materials Figure S1.

The structural transformation of the YRD patent cooperation network is visualized in Figure 1. Figure 1a illustrates the early evolution from 2005 to 2020, showing a clear transition from a fragmented core–periphery structure to a polycentric configuration. Figure 1b presents the 2021–2022 period, during which the network continued to densify. Figure 1c and Figure 1d provide enlarged views of the densest years, 2023 and 2024, respectively. For 2024, only the top 30 strongest collaborative ties are displayed to maintain visual clarity (see Supplementary Materials for the full network). The evolutionary stages are summarized in Table 4.

Stage I: Node Function Polarization (2005–2010). This stage is characterized by a typical core–periphery structure with Shanghai as the dominant core. As shown in Figure 1a, the 2005 network is highly fragmented, with a small number of core nodes forming dense connections while most peripheral nodes remain isolated. Network density increased from 0.052 to 0.190 during this period, while fragmentation decreased from 68.3% to 14.3% (Table 3). Shanghai maintained the highest degree centrality, reflecting its role as the primary hub. By 2010 [Figure 1b], the network shows significant integration, with more nodes becoming connected and the emergence of multiple cores alongside Shanghai (Figure 1a, 2005 and 2010 panels). This stage marks the critical transition from a single-core to a multi-core structure.

Stage II: Cluster Structure Emergence (2010–2015). A critical transition occurred in this stage. Network density increased to 0.4415, and fragmentation dropped to 7.2%, indicating near-complete integration. The average clustering coefficient decreased markedly while the average path length shortened, indicating that the network had become highly connected but maintained a hub-dominated architecture, with a small number of core cities (Shanghai, Nanjing, Hangzhou, Hefei) serving as primary connectors. Figure 1c illustrates the 2015 network, which exhibits clear clustering patterns. Geographically proximate clusters emerge, notably the “Shanghai–Suzhou–Wuxi–Changzhou” cluster in Jiangsu and the “Hangzhou–Ningbo–Shaoxing” cluster in Zhejiang. Crucially, Nanjing and Hefei emerged as critical bridges connecting different clusters, a pattern visually evident (Figure 1a, 2015 panel).

Stage III: Network System Restructuring (2015–2020). In this stage, the network matured into a polycentric, integrated structure. Network density reached 0.548 in 2020, and average weighted degree increased to 673.41, indicating extensive inter-city connections. The average clustering coefficient remained low (0.0095–0.0113), while the path length decreased to 1.45, demonstrating efficient connectivity. As depicted in Figure 1a (2020 panel), the 2020 network exhibits a balanced configuration with multiple core nodes—Shanghai, Nanjing, Hangzhou, Hefei, Wuxi, and Ningbo—each serving as a regional hub. Notably, Anhui cities, particularly Hefei and Wuhu, became fully integrated into the network, serving as important connectors between the western region and the YRD core (Figure 1a, 2020 panel).

Stage IV: Highly Integrated Network (2021–2024). This stage represents the continuation and consolidation of the polycentric integrated structure. Network density remained stable between 0.182 and 0.220, and the average binary degree fluctuated around 7.3–8.8, indicating sustained high levels of inter-city cooperation. Fragmentation fell further to 4.9% during 2021–2023 and reached 0% in 2024, meaning that all 41 cities became fully connected in a single component. The weighted average clustering coefficient remained low, and the average path length stayed at 1.9–2.0, confirming the persistence of a hub-dominated topology with efficient global connectivity. Notably, the network achieved full connectivity in 2024, reflecting a mature and highly resilient innovation ecosystem (Figure 1b for 2021–2022; Figure 1c,d for 2023–2024). For 2024, the network is displayed in Figure 1d with only the top 30 strongest collaborative ties (out of 180+ total edges) to highlight the most significant inter-city partnerships and ensure visual clarity. The complete 2024 network, including all edges, is provided in Supplementary Materials (Figure S1). This filtering strategy is consistent with established practices in visualizing dense innovation networks.

### 3.3. Node Centrality Evolution

Table 5 presents the degree centrality rankings of cities in the YRD patent cooperation network. Shanghai consistently ranked among the top three throughout the study period and maintained the highest degree centrality from 2021 to 2024. Its absolute degree values increased substantially after 2020, reflecting the growing volume of cooperative patents. Nanjing emerged as a core node, ranking first in degree centrality in 2010, 2015, and 2020. Wuxi also demonstrated strong centrality, ranking second in 2020 and remaining among the top five in most years. Hefei showed a remarkable increase after 2010, reaching the second-highest degree in 2021 and 2024, demonstrating the deepening integration of Anhui province into the regional innovation network [8,9]. Suzhou (Jiangsu) also exhibited strong performance, ranking among the top three from 2021 to 2023.

### 3.4. Structural Hole Evolution

Structural hole indicators measure the extent to which cities occupy bridging positions that confer information advantages [22]. Table 6 presents constraint and efficiency scores for each core city, with lower constraint values indicating stronger structural hole advantages.

Shanghai consistently recorded the lowest constraint scores across the entire study period, decreasing from 0.205 in 2005 to 0.182 in 2024. This finding confirms its superior and persistent bridging position. Nanjing also maintained a strong bridging role before 2020, with constraint scores below 0.25 from 2005 to 2020. However, its constraint increased notably between 2021 and 2024, ranging from 0.707 to 0.952. These fluctuations may reflect temporary shifts in collaboration patterns or data limitations. Hangzhou and Hefei experienced declining constraint after 2010. Hefei’s constraint dropped from 0.333 in 2005 to 0.236 in 2024, demonstrating its emergence as an important bridging city. Wuhu underwent a dramatic reduction from 1.000 in 2005 to 0.205 in 2020, confirming its integration into the network, although its constraint increased slightly to 0.508 in 2024.

Efficiency scores, which capture information benefits per connection, remained high for core cities. Shanghai, Hefei, and Wuhu all achieved efficiency scores above 0.84 in 2024.

### 3.5. Resilience Assessment Results

To evaluate the network’s ability to withstand disruptions, we simulated targeted attacks by removing nodes in descending order of their weighted degree centrality [23,25]. All simulations were performed using Python 3.14 with the NetworkX library (version 3.6.1) and NumPy (version 2.4.4). The procedure for each network was as follows:

First, constructing a weighted undirected graph from the 41 × 41 adjacency matrix, where edge weights represent the number of cooperative patents between two cities. Second, computing the weighted degree of every node and sorted nodes in descending order. Third, sequentially removing nodes according to this order. After each removal, we calculate the size of the largest connected component and divide it by the original total number of nodes, which is 41, to obtain the proportion of the surviving largest component R(f). Fourth, recording the removal fraction f, i.e., the cumulative number of removed nodes divided by 41, along with the corresponding R(f). Fifth, plotting a robustness curve with f on the x-axis ranging from 0 to 1 and R(f) on the y-axis. Finally, computing the resilience index *Ω* as the area under this curve using the trapezoidal rule as implemented in the numpy.trapezoid function.

The resulting resilience indices are reported in Table 7. The resilience index increased from 0.0836 in 2005 to 0.3572 in 2010, and reached 0.5000 in 2015, remaining at this level through 2020 (Table 7). For 2021–2024, Ω fluctuated between 0.4057 and 0.4485, with the highest value of 0.4485 recorded in 2023. Although these values are lower than the 2015–2020 peak, they remain substantially above the 2005 baseline, suggesting that the network maintained a moderate level of robustness after its structural consolidation.

Table 8 reports the detailed results of core node removal simulations. Following the same procedure described in Section 3.5, we simulated targeted attacks. For each year’s weighted undirected network, we computed weighted degree centrality, removed nodes in descending order, and recorded the size of the largest connected component after each removal. The table shows how the largest component declines as core nodes are removed sequentially.

A comparison across years reveals a clear shift in network vulnerability. In 2005, removing Shanghai alone reduced the largest connected component from 23 to 18, a reduction of 21.7% (Table 8). By 2010, removing the top-ranked node (Nanjing) reduced the component from 38 to 36, a drop of 5.3%. In 2015, removing four core nodes—Nanjing, Hangzhou, Shanghai, and Ningbo—together lowered the component from 41 to 37, a decline of 9.8%. This stark difference shows that the multi-center structure that had taken shape by 2015 substantially improved the network‘s resilience to the failure of its most central nodes [14].

For 2021 to 2024, the initial largest component contained 40 cities because Zhoushan remained isolated until 2024. Removing Shanghai alone reduced the component to 38 or 39, similar to the pattern after 2010. In 2024, the network became fully connected with 41 nodes, and deleting Shanghai together with Hefei, Suzhou_js, and Nantong lowered the component from 41 to 35, a decline of 14.6% (Table 8).

The resilience assessment therefore offers strong evidence that moving from a single-core to a multi-center configuration does more than improve connectivity and efficiency; it also substantially strengthens the network’s ability to withstand targeted attacks on its most influential nodes [14].

### 3.6. Correlation Between Structural Characteristics and Resilience

To examine the relationship between network structural characteristics and resilience, we calculated Pearson correlation coefficients between five structural indicators and the resilience index (Table 9). All coefficients are based on eight time points from 2005 to 2024.

Weighted-degree share entropy showed the strongest positive correlation with the resilience index (r = 0.9824, *p* < 0.001, 95% CI [0.903, 0.997]), suggesting that the redistribution of innovation activity across cities is closely associated with systemic robustness. Density also exhibited a strong positive correlation (r = 0.7456, *p* = 0.0337, 95% CI [0.086, 0.951]), as did average degree (r = 0.7926, *p* = 0.0190, 95% CI [0.199, 0.961]) and clustering coefficient (r = 0.7801, *p* = 0.0224, 95% CI [0.168, 0.958]). In contrast, average path length showed a strong negative correlation (r = −0.7368, *p* = 0.0371, 95% CI [−0.949, −0.067]), indicating that shorter path lengths are associated with higher resilience in this hub-dominated network.

To assess the robustness of these correlations given the limited sample size (*n* = 8), we conducted two supplementary analyses. First, Spearman rank correlations confirmed the positive association between weighted-degree share entropy and resilience (r = 0.8862, *p* = 0.0034), though associations for other indicators (e.g., clustering coefficient, r = 0.4671, *p* = 0.2433) were not significant in the rank-based test. Second, first-difference correlations (year-to-year changes) revealed that weighted-degree share entropy remained strongly associated with changes in resilience (r = 0.9049, *p* = 0.0051), while other indicators showed weaker first-difference associations (Table 9). This suggests that the observed relationship between weighted-degree share entropy and resilience is not purely driven by common time trends, whereas the associations for density, average degree, and path length may partly reflect shared temporal trends.

Given the limited number of time points (*n* = 8), all correlations should be interpreted as temporal co-variation rather than causal relationships. The strong associations suggest that the redistribution of innovation activity and the improvement of resilience occurred concurrently, but do not establish a directional causal link.

### 3.7. Weighted-Degree Share Entropy over Time

To formally quantify the “centralized-to-distributed” transition, we computed the Shannon entropy of the weighted-degree share distribution for each year: $H=-\sum_{k=1}^{N}p_{k}lnp_{k}$, where $p_{k}=d_{k}/\sum_{j}d_{j}$, where $d_{k}$ is the weighted degree of city *k*, and *N* = 41 is the total number of cities.

The results are reported in the last column of Table 2. *H* increased from 2.999 in 2005 to 3.645 in 2020, indicating that innovation activity became increasingly distributed across cities over time. The strong correlation between weighted-degree share entropy and resilience (r = 0.9822, *p* = 0.000014) suggests that the redistribution of innovation activity is closely associated with enhanced systemic robustness, although this relationship should be interpreted with caution given the limited number of time points (*n* = 8).

## 4. Discussion

This study has examined the structural evolution and resilience of the Yangtze River Delta patent cooperation network over two decades. The findings reveal a clear four-stage trajectory: from an initial hub-dominated configuration, through the formation of collaborative clusters and systemic restructuring, to full integration by 2024. This pattern of deepening connectivity and the transition toward a polycentric architecture is consistent with the broader literature on regional innovation network development [10]. From a sustainability perspective, what makes these results particularly relevant is the demonstration that a region’s innovation system can become more robust not simply by adding more connections, but by reconfiguring how those connections are distributed.

Sustainability in a regional context encompasses not only environmental outcomes but also the long-term adaptive capacity of socio-economic systems. A region’s ability to sustain innovation under disruption is therefore an integral component of its sustainability profile. From this perspective, the resilience of the YRD’s patent cooperation network is not merely a technical issue of network topology; it is a measure of how well the region’s innovation system can withstand shocks and continue to function over time.

### 4.1. Polycentricity as a Foundation for Sustainable Regional Innovation

The network’s growing resilience over the study period is most clearly evidenced in the targeted attack simulations. In 2010, removing the top-ranked node (Nanjing) reduced the largest connected component from 38 to 36, a drop of 5.3% (Table 8). By 2015, removing four core nodes—Nanjing, Hangzhou, Shanghai, and Ningbo—together lowered the component from 41 to 37, a decline of 9.8%. In 2020, removing four core nodes (Nanjing, Wuxi, Hangzhou, and Shanghai) produced a reduction from 41 to 37, a decline of 9.8%, indicating that the network maintained its resilience level after the structural consolidation in 2015.This shift from high vulnerability to a single point of failure to substantially greater tolerance of multiple node losses provides strong empirical evidence consistent with the view that a polycentric network architecture is associated with systemic robustness. From a network topology perspective, this transition can be interpreted as a redistribution of innovation activity: concentration in a single hub creates a fragile configuration, whereas a distributed multi-hub structure spreads risk and enhances robustness against localized disruptions. The degree distribution entropy metric offers quantitative support for this redistribution process.

The implication for sustainable regional development is straightforward: relying on a single innovation hub creates a fragile system, while a distributed polycentric structure spreads risk and makes the network more resilient to local disruptions. The same logic applies at a broader policy level. A region that concentrates its innovation capacity in one dominant city faces a sustainability risk if that hub is weakened by economic downturn, policy shift, or external shock. The YRD’s evolution toward a polycentric structure demonstrates that resilience can be built incrementally by fostering secondary hubs, particularly in less central provinces. Anhui’s cities, notably Hefei and Wuhu, have moved from peripheral positions to become important bridging nodes. Their integration into the core network illustrates how targeted connectivity can reduce regional disparity and contribute to a more balanced and durable innovation landscape.

Interestingly, the YRD patent cooperation network exhibits a clear small-world property (σ>1 for all years after 2010), as confirmed by comparison with random networks of equivalent size and edge count (see Appendix A Table A1). This suggests that the network combines efficient global connectivity through its core hubs with moderate local clustering, a topology that balances knowledge diffusion efficiency with local redundancy. Instead, it displays a hub-and-spoke topology: a small number of core cities maintain direct connections to a large number of peripheral cities. This structure arises because patent cooperation is driven by a few major innovation hubs (Shanghai, Nanjing, Hangzhou, Hefei) rather than distributed local clusters. This topology has important implications for resilience: while hub-and-spoke networks are efficient for knowledge diffusion, they can be vulnerable to the failure of core hubs—a vulnerability that the YRD has mitigated by developing multiple co-existing hubs (polycentricity), even though the overall structure remains hub-dominated.

This finding aligns with the observed resilience patterns: the emergence of multiple co-existing hubs by 2015 explains why the network became more tolerant to the removal of individual core nodes, even without developing dense local clustering. The resilience gain comes not from redundant local connections but from the diversification of hub functions across multiple cities.

The visual progression from a Shanghai-dominated network in 2005 (Figure 1a) to a polycentric configuration by 2020 (Figure 1a), and further to the dense, multi-hub structure in 2023–2024 (Figure 1c,d), corroborates the quantitative resilience assessment. Despite the increasing density, the network maintains a hub-dominated topology with multiple co-existing cores.

### 4.2. Structural Drivers of Resilience and Hub-Dominated Topology

The correlation analysis reveals strong temporal associations between several structural features and resilience. Density, average degree, and degree entropy all exhibit strong positive correlations with resilience over the study period, while average path length shows a moderate negative association. However, with only eight time points, these correlations may partly reflect common time trends rather than purely structural mechanisms. Future research with more frequent observations or panel data would be needed to establish causal relationships.

To formally assess the network’s topological nature, we computed the small-world coefficient σ following Humphries and Gurney (2008) [26]. For each year from 2010 onward, we generated 500 Erdős–Rényi random networks with the same number of nodes and edges as the observed network, and calculated the average clustering coefficient Crand and average shortest path length Lrand. As detailed in Appendix A Table A1, σ>1 for all years from 2010 to 2024, ranging from 1.3226 in 2020 to 2.7730 in 2021, indicating that the network exhibits clear small-world characteristics. This finding does not contradict the hub-dominated nature of the network; rather, it suggests that the network combines efficient global connectivity through its core hubs (Shanghai, Nanjing, Hangzhou, Hefei) with moderate local clustering.

This topology arises because patent cooperation is driven by a few major innovation hubs rather than distributed local clusters. In this hub-dominated network, the relatively low local clustering means that resilience is not derived primarily from local redundancy, as in classical small-world systems, but from the diversification of hub functions across multiple co-existing hubs. This is precisely the mechanism we observe: the emergence of multiple hubs distributes risk, so that the failure of any single hub does not critically fragment the system.

The stable but lower resilience levels observed from 2021 to 2024, relative to the 2015 peak, suggest that adding more connections beyond a certain point may yield diminishing returns for robustness. Full connectivity, achieved in 2024, did not automatically restore the resilience peak of 2015. This implies that network architecture matters as much as network density. A fully connected network can still be vulnerable if too many pathways depend on a small set of critical nodes. Monitoring the distribution of bridging ties and identifying potential bottlenecks should therefore be part of any long-term resilience strategy. From a network architecture standpoint, this finding suggests that increasing the distribution of innovation activity beyond an optimal level—by adding redundant connections that do not fundamentally alter the core–periphery structure—may not yield proportional gains in systemic robustness.

### 4.3. Comparison with Related Networks and Implications for Sustainability

The three-stage evolution identified in earlier YRD studies is extended here to a fourth stage of full integration. A more revealing comparison, however, lies with other types of networks. Research on tourism economic networks, for instance, has found that those networks exhibit what has been called “strong recovery but weak resistance”—they can bounce back after disruption but are easily disrupted in the first place [27]. Patent cooperation networks, by contrast, appear to develop strong resistance through structural redundancy and polycentric configuration. The difference likely stems from the nature of the relationships involved. Tourism flows are highly sensitive to external shocks and have low entry barriers, making them volatile. Patent collaborations are built on longer-term strategic commitments, formal agreements, and repeated interactions, which embed redundancy into the network.

This distinction has direct relevance for sustainability policy. For a region seeking to maintain its innovation capacity over decades, building resilient structures is not the same as building large structures. A high volume of transient, low-commitment collaborations may not produce the same protective effect as a smaller but more deeply embedded set of relationships that provide alternative pathways when some nodes fail. The YRD’s experience suggests that resilience is not an incidental by-product of growth, it is a design feature that can be actively cultivated through network architecture.

### 4.4. Policy Implications

Several implications follow for regional innovation policy.

Foster polycentric development. Policies should continue to support secondary innovation hubs, particularly in provinces such as Anhui, where cities like Hefei and Wuhu already serve as important bridges between peripheral areas and the core region. Spreading innovation capacity across multiple centers reduces the risk that a single disruption could paralyze the entire system.

Leverage structural redundancy. While clustering alone shows a limited direct association with resilience in this hub-dominated network, the strong correlations of density and degree entropy with resilience suggest that fostering dense, multi-lateral collaborations and diversifying innovation hubs can enhance systemic robustness. This can be achieved through cross-city research consortia, shared technology platforms, and funding schemes that require collaboration among three or more partners rather than only bilateral arrangements.

Monitor network evolution continuously. The slight decline in resilience from 2015 to 2020 and the stable but lower levels from 2021 to 2024 indicate that resilience is not monotonic. Regular network assessments using the metrics developed here—density, clustering coefficient, path length, and resilience index—can help policymakers identify emerging vulnerabilities, such as over-reliance on a small number of bridging nodes, before they become critical.

Integrate peripheral cities through targeted connectivity. For less connected cities in northern Jiangsu and western Anhui, policies should focus on building direct links to core hubs. Innovation corridors, incentives for joint patent applications with leading cities, and investment in digital infrastructure to lower coordination costs can accelerate their integration and contribute to more balanced regional development.

### 4.5. Theoretical Contributions

This study makes three contributions to the literature on innovation network resilience.

First, it provides empirical evidence that full network connectivity does not guarantee maximal resilience. The Yangtze River Delta patent cooperation network achieved complete integration of all 41 cities in 2024, yet its resilience index remained substantially lower than the 2015 peak, when the network was less connected but exhibited a more robust polycentric configuration. This counter-intuitive finding challenges the implicit assumption that higher connectivity necessarily yields stronger resilience, suggesting instead that network architecture, and specifically the distribution of innovation activity across multiple hubs, matters as much as its sheer volume.

Second, it reveals a potential diminishing-returns effect of hub-diversification on resilience. Between 2005 and 2020, the degree entropy of the network increased from 2.999 to 3.645, while the resilience index peaked in 2015 and then stabilized or even declined. After 2020, further increases in connectivity did not produce corresponding gains in resilience. This pattern points to the possible existence of an optimal level of hub diversification beyond which additional connectivity yields diminishing resilience returns, a phenomenon that has received little attention in the innovation network literature.

Third, it offers a methodological caution about the use of patent data for real-time resilience assessment. This finding also serves as a methodological caveat: interpretations of network resilience must be grounded in data integrity. Incomplete patent records—particularly for recent years subject to publication lags, can produce artificial network fragmentation that resembles structural vulnerability. Distinguishing between genuine topological fragility and artifacts of data incompleteness is therefore essential for valid resilience assessment. This underscores the imperative of rigorous data validation and transparent reporting of data limitations in innovation network studies.

Together, these findings call for a more nuanced understanding of network resilience that goes beyond density and connectivity. They suggest that resilience is not a monotonic function of connectivity or clustering, and that the relationship between network structure and resilience may involve thresholds, trade-offs, and diminishing returns. From a network architecture perspective, the YRD network’s evolution from a concentrated to a distributed configuration demonstrates that increasing the distribution of innovation activity within a bounded system is associated with enhanced topological resilience—but only up to a certain point, beyond which additional distribution may yield diminishing marginal returns.

## 5. Conclusions

This study has investigated the structural evolution and topological resilience of the Yangtze River Delta (YRD) patent cooperation network from 2005 to 2024. The findings reveal a fundamental transformation: the network has evolved from a fragmented, single-core configuration dominated by Shanghai into a fully integrated, polycentric architecture. The degree distribution entropy increased from 2.999 in 2005 to 3.645 in 2020, quantitatively confirming the progressive redistribution of innovation activity across the region.

The polycentric structure that had taken shape by 2015 is substantially associated with network resilience. This increased robustness corresponds to a transition from a fragile state—where most innovation capacity is concentrated in one node—to a more distributed state that spreads risk and eliminates single points of failure, as quantitatively reflected in the increasing weighted-degree share entropy over time. Targeted attack simulations show that the network’s ability to withstand core node failures improves markedly as the configuration shifts from single-core to multi-center. Notably, full connectivity achieved in 2024 did not restore the peak resilience observed in 2015, suggesting that beyond an optimal level, further increases in network entropy may yield diminishing returns for systemic robustness. Correlation analysis reveals strong temporal associations between resilience and density, average degree, degree entropy, and average path length. These associations suggest that the structural evolution of the network toward polycentricity co-occurred with improvements in resilience, although the limited number of time points (*n* = 8) precludes strong causal claims.

This study makes three theoretical contributions. First, it introduces a network resilience perspective to the study of inter-city innovation networks, extending concepts of robustness from urban and ecological systems to collaborative innovation, and provides quantitative evidence that the redistribution of innovation activity—as measured by degree distribution entropy—is associated with enhanced system robustness against targeted attacks. Second, it constructs an integrated “structure–node function–resilience” analytical framework that captures temporal dynamics, offering a replicable methodology for future research, within which entropy serves as a unifying metric to quantify polycentricity and its relationship with robustness. Third, it provides empirical evidence consistent with the view that polycentric configurations are associated with network robustness, and reveals that full connectivity alone does not guarantee maximal resilience, a counter-intuitive finding that challenges the assumption that more connections always yield stronger robustness and suggests the existence of an optimal level of distribution in innovation activity beyond which additional connectivity yields diminishing returns.

Several limitations of this study should be acknowledged. First, the patent cooperation data capture only formal collaborative outputs, potentially overlooking informal knowledge exchanges that may also contribute to network resilience. Second, while we have introduced degree distribution entropy as a quantitative metric for measuring the spatial distribution of innovation activity, this captures only one dimension of network topology; future work could extend the analysis to include other entropy-based measures, such as community entropy or structural entropy, to provide a more comprehensive assessment of network configuration and resilience.

Several directions for future research emerge from this study. Extending the analysis to include continuous time series data would allow a finer-grained examination of entropy trajectories and could reveal additional transition points. Incorporating random attack simulations and recovery dynamics would provide a more complete assessment of network resilience by distinguishing between resistance and recovery. Integrating network resilience metrics into econometric models could test whether more resilient, i.e., higher-entropy, network structures lead to superior innovation outputs, such as improved patent quality or regional economic growth. Comparative studies across other urban agglomerations, such as the Beijing–Tianjin–Hebei region and the Guangdong–Hong Kong–Macao Greater Bay Area, would help validate the theoretical findings and assess their broader applicability. Combining quantitative network analysis with qualitative case studies could uncover the causal processes and institutional mechanisms that drive entropy redistribution. Finally, addressing the issue of data incompleteness—for example through real-time patent tracking, the use of supplementary data sources, e.g., co-publications or R&D project collaborations, or statistical imputation techniques—would improve the reliability of resilience assessments for recent years.

## Figures and Tables

**Table 1:** Study area: 41 cities in the Yangtze River Delta urban agglomeration.

Province/Municipality	City
Shanghai	Shanghai
Jiangsu	Nanjing, Wuxi, Xuzhou, Changzhou, Suzhou_js, Nantong, Lianyungang, Huai’an, Yancheng, Yangzhou, Zhenjiang, Taizhou_js, Suqian
Zhejiang	Hangzhou, Ningbo, Wenzhou, Jiaxing, Huzhou, Shaoxing, Jinhua, Quzhou, Zhoushan, Taizhou_zj, Lishui
Anhui	Hefei, Wuhu, Bengbu, Huainan, Ma’anshan, Huaibei, Tongling, Anqing, Huangshan, Chuzhou, Fuyang, Suzhou_ah, Lu’an, Bozhou, Chizhou, Xuancheng

Note: The study area comprises 41 prefecture-level and above cities as defined in the Outline of the Yangtze River Delta Regional Integration Development Plan issued by the State Council of China in December 2019.

**Table 2:** Overall characteristics of the Yangtze River Delta patent cooperation network (2005–2024).

Year	Density	Average Degree	Clustering Coefficient (Weighted)	Average Path Length	Degree Entropy
2005	0.0524	5.56	0.0177	2.1858	2.9987
2010	0.1902	52.63	0.0394	1.8791	3.3476
2015	0.4415	282.98	0.0113	1.5634	3.5986
2020	0.5476	673.41	0.0095	1.4524	3.6452
2021	0.1817	174.44	0.0118	2.0295	3.4244
2022	0.2122	186.83	0.0118	1.9205	3.4384
2023	0.2037	193.66	0.0125	1.9436	3.4542
2024	0.2195	193.02	0.0107	1.9537	3.4735

Note: All networks are undirected and weighted. Density and average path length are computed on binary projections of the weighted adjacency matrices. The “Clustering Coefficient (Weighted)” column reports the weighted clustering coefficient computed using the Barrat et al. (2004) [18] formula (Equation (4) in Section 2.3.2). This weighted metric captures both the number and intensity of connections among a node’s neighbors and may yield values greater than 1. The binary clustering coefficients used for small-world analysis (Appendix A Table A1) are computed on the binary projections of the networks and range between 0 and 1. Weighted-degree share entropy is the Shannon entropy of the weighted-degree share distribution, $H=-\sum p_{k}lnp_{k}$, where $p_{k}=d_{k}/\sum_{j}d_{j}$, $d_{k}$ is the weighted degree of city *k*, and the summation is over all 41 cities. Higher values indicate more distributed configurations.

**Table 3:** Network connectivity and fragmentation (2005–2024).

Year	Connectedness	Fragmentation	Number of Components
2005	0.317	0.683	15
2010	0.857	0.143	4
2015	0.928	0.072	4
2020	0.928	0.072	4
2021	0.951	0.049	2
2022	0.951	0.049	2
2023	0.951	0.049	2
2024	1.000	0.000	1

Note: Connectedness is the proportion of nodes in the largest connected component. Fragmentation is defined as 1-connectedness. The number of connected components is the total count of isolated and clustered components in each year.

**Table 4:** Evolutionary stages of the Yangtze River Delta patent cooperation network (2005–2024).

Stage	Period	Key Characteristics
Stage I	2005–2010	Node function polarization; core–periphery structure; Shanghai dominant
Stage II	2010–2015	Cluster structure emergence; hub-dominated architecture emerges; multiple cores develop
Stage III	2015–2020	Network system restructuring; multi-center integration; optimal connectivity
Stage IV	2021–2024	Highly integrated network; full connectivity achieved (2024); hub-dominated topology consolidated

Note: Stage characteristics are synthesized from the network metrics reported in Table 2 and Table 3 and the visual evidence presented in Figure 1.

**Table 5:** Degree centrality rankings of core cities (2005–2024).

Rank	2005	Degree	2010	Degree	2015	Degree	2020	Degree
1	Shanghai	69	Nanjing	249	Nanjing	1278	Nanjing	3036
2	Ningbo	29	Shanghai	233	Hangzhou	1149	Wuxi	2680
3	Hangzhou	16	Hangzhou	221	Shanghai	1122	Hangzhou	2580
4	Nanjing	14	Ningbo	213	Ningbo	1116	Shanghai	2486
5	Suzhou_js	10	Wuxi	153	Wuxi	893	Ningbo	2276
6	Huai’an	10	Suzhou_js	119	Lishui	587	Suzhou_js	1299
7	Wenzhou	10	Changzhou	92	Taizhou_zj	572	Nantong	1178
8	Wuxi	9	Shaoxing	89	Suzhou_js	455	Lishui	1161
9	Changzhou	7	Jinhua	75	Nantong	440	Taizhou_zj	1158
10	Jinhua	7	Yangzhou	72	Xuzhou	414	Wenzhou	839
**Rank**	**2021**	**Degree**	**2022**	**Degree**	**2023**	**Degree**	**2024**	**Degree**
1	Shanghai	2283	Shanghai	2456	Shanghai	2577	Shanghai	2412
2	Hefei	718	Suzhou_js	745	Suzhou_js	828	Hefei	840
3	Suzhou_js	627	Hefei	696	Hefei	702	Suzhou_js	756
4	Wuxi	540	Wuxi	602	Nantong	589	Nantong	487
5	Zhenjiang	504	Nantong	511	Wuxi	477	Wuxi	479
6	Bengbu	426	Bengbu	383	Bengbu	359	Bengbu	385
7	Nantong	382	Zhenjiang	342	Zhenjiang	321	Zhenjiang	308
8	Nanjing	379	Nanjing	247	Changzhou	272	Nanjing	303
9	Changzhou	202	Jiaxing	214	Nanjing	235	Changzhou	275
10	Jiaxing	144	Changzhou	188	Jiaxing	203	Jiaxing	219

Note: Degree values are weighted degree (sum of cooperative patent counts) computed on undirected weighted networks for all years. All values have been recalculated using a unified city-name matching protocol to ensure cross-year consistency. Data sources for 2005–2020 and 2021–2024 differ but were validated for consistency using the overlapping year 2020 (see Section 2.2).

**Table 6:** Structural hole indicators for core cities (2005–2024).

City	2005	2010	2015	2020	2021
**Constraint**					
Shanghai	0.205	0.257	0.204	0.220	0.193
Nanjing	0.247	0.223	0.172	0.178	0.707
Hangzhou	0.195	0.251	0.279	0.250	1.034
Hefei	0.333	0.283	0.247	0.210	0.370
Wuhu	1.000	0.368	0.242	0.205	0.973
**Efficiency**					
Shanghai	0.971	0.867	0.869	0.883	0.902
Nanjing	0.905	0.851	0.884	0.912	0.562
Hangzhou	1.000	0.813	0.861	0.897	0.390
Hefei	1.000	0.878	0.829	0.862	0.941
Wuhu	1.000	0.895	0.860	0.861	0.510
**City**	**2022**	**2023**	**2024**		
**Constraint**					
Shanghai	0.190	0.184	0.182		
Nanjing	0.830	0.952	0.879		
Hangzhou	0.707	0.652	0.786		
Hefei	0.316	0.276	0.236		
Wuhu	0.529	0.418	0.508		
**Efficiency**					
Shanghai	0.903	0.891	0.916		
Nanjing	0.529	0.446	0.486		
Hangzhou	0.597	0.749	0.611		
Hefei	0.936	0.937	0.944		
Wuhu	0.817	0.824	0.870		

Note: All indicators were computed using NetworkX’s structural holes algorithm on undirected weighted networks.

**Table 7:** Resilience indices under targeted attacks (2005–2024).

Year	Resilience Index (Ω)
2005	0.0836
2010	0.3572
2015	0.5000
2020	0.5000
2021	0.4358
2022	0.4057
2023	0.4485
2024	0.4396

Note: All resilience indices were computed using the corrected node-removal simulation protocol described in Section 2.4.1. The indices for 2021–2024 are based on annual network simulations. All values have been fully recalculated from the original weighted matrices.

**Table 8:** Impact of core node removal on the largest connected component.

Year	Removed Nodes	f	$C_{\max}$	R(f)
2005	—	0.000	23	0.561
	Shanghai	0.024	18	0.439
	Shanghai, Ningbo	0.049	15	0.366
	Shanghai, Ningbo, Hangzhou	0.073	10	0.244
	Shanghai, Ningbo, Hangzhou, Nanjing	0.098	4	0.098
2010	—	0.000	38	0.927
	Nanjing	0.024	36	0.878
	Nanjing, Shanghai	0.049	35	0.854
	Nanjing, Shanghai, Hangzhou	0.073	33	0.805
	Nanjing, Shanghai, Hangzhou, Ningbo	0.098	32	0.780
2015	—	0.000	41	1.000
	Nanjing	0.024	40	0.976
	Nanjing, Hangzhou	0.049	39	0.951
	Nanjing, Hangzhou, Shanghai	0.073	38	0.927
	Nanjing, Hangzhou, Shanghai, Ningbo	0.098	37	0.902
2020	—	0.000	41	1.000
	Nanjing	0.024	40	0.976
	Nanjing, Wuxi	0.049	39	0.951
	Nanjing, Wuxi, Hangzhou	0.073	38	0.927
	Nanjing, Wuxi, Hangzhou, Shanghai	0.098	37	0.902
2021	—	0.000	40	0.976
	Shanghai	0.024	38	0.927
	Shanghai, Hefei	0.049	36	0.878
	Shanghai, Hefei, Suzhou_js	0.073	35	0.854
	Shanghai, Hefei, Suzhou_js, Wuxi	0.098	34	0.829
2022	—	0.000	40	0.976
	Shanghai	0.024	39	0.951
	Shanghai, Suzhou_js	0.049	38	0.927
	Shanghai, Suzhou_js, Hefei	0.073	36	0.878
	Shanghai, Suzhou_js, Hefei, Wuxi	0.098	35	0.854
2023	—	0.000	40	0.976
	Shanghai	0.024	39	0.951
	Shanghai, Suzhou_js	0.049	38	0.927
	Shanghai, Suzhou_js, Hefei	0.073	36	0.878
	Shanghai, Suzhou_js, Hefei, Nantong	0.098	34	0.829
2024	—	0.000	41	1.000
	Shanghai	0.024	40	0.976
	Shanghai, Hefei	0.049	37	0.902
	Shanghai, Hefei, Suzhou_js	0.073	36	0.878
	Shanghai, Hefei, Suzhou_js, Nantong	0.098	35	0.854

Note: *f* is the cumulative fraction of nodes removed (number removed/41). $C_{\max}$ is the size of the largest connected component after removal, and $R(f)=C_{\max}/41$ measures network robustness. The denominator is fixed at 41 (total number of cities) for all years to ensure cross-year comparability. The node removal order strictly follows the weighted degree rankings shown in Table 5 for each year. For 2021–2023, the initial largest component contained 40 nodes (Zhoushan was isolated until 2024), giving *R*(0) = 40/41 = 0.976.

**Table 9:** Correlation matrix of network structural characteristics and resilience index.

Indicator	Pearson r	*p*-Value	95% CI	Spearman r	*p*-Value	First-Difference r	*p*-Value
**Density**	0.7456	0.0337	[0.086, 0.951]	0.8264	0.0114	0.6274	0.1315
**Average Degree**	0.7926	0.0190	[0.199, 0.961]	0.6826	0.0621	0.4095	0.3616
**Clustering Coefficient**	0.7801	0.0224	[0.168, 0.958]	0.4671	0.2433	0.2862	0.5337
**Average Path Length**	−0.7368	0.0371	[−0.949, −0.067]	−0.8024	0.0165	−0.6541	0.1109
**Weighted-Degree Share Entropy**	0.9824	0.000013	[0.903, 0.997]	0.8862	0.0034	0.9049	0.0051

Note: Correlations are calculated from eight time points (2005, 2010, 2015, 2020, 2021–2024). Pearson r and 95% confidence intervals (CI) are reported. Spearman rank correlations and first-difference correlations (year-to-year changes) are provided as robustness checks. The first-difference results show that the association between weighted-degree share entropy and resilience remains strong (r = 0.9049, *p* = 0.0051) even after accounting for common time trends, while other indicators show weaker or non-significant first-difference correlations, suggesting that their observed associations may partly reflect shared temporal trends. All significance tests are two-tailed.

## Data Availability

The patent data for the period 2005–2020 used in this study are publicly available from the National Earth System Science Data Center (http://www.geodata.cn) (accessed on 24 March 2026), under the dataset “Yangtze River Delta urban agglomeration inter-city patent cooperation network data (2001–2020)”. The raw patent data for 2021–2024 were retrieved from the China National Intellectual Property Administration (CNIPA) Patent Search and Analysis System (https://pss-system.cponline.cnipa.gov.cn/) (accessed on 26 March 2026), which is a free public service. The aggregated weighted adjacency matrices for each year (2005, 2010, 2015, 2020, and 2021–2024) derived from these sources, as well as the network analysis results, are openly available in the Zenodo repository at https://doi.org/10.5281/zenodo.20459833. No additional proprietary or restricted data were used in this study.

## Supplementary Materials

The following supporting information can be downloaded at: https://www.mdpi.com/article/10.3390/e28070826/s1, Figure S1: Yangtze River Delta Patent Cooperation Network (2024).

## Abbreviation

The following abbreviation is used in this manuscript:

YRD	The Yangtze River Delta

## Appendix A

**Table A1:** Small-world properties of the YRD patent cooperation network (2005–2024).

Year	C	Crand	L	Lrand	σ	Random Networks Used
2005	—	—	—	—	—	—
2010	0.5235	0.2217	1.8791	1.9001	2.3878	498
2015	0.6409	0.4410	1.5634	1.5586	1.4486	500
2020	0.7239	0.5474	1.4524	1.4524	1.3226	500
2021	0.5367	0.1912	2.0295	2.0050	2.7730	498
2022	0.5744	0.2221	1.9205	1.8848	2.5379	499
2023	0.5481	0.2128	1.9436	1.9140	2.5363	499
2024	0.4930	0.2186	1.9537	1.8903	2.1824	500

Note: *C* and *L* denote the average clustering coefficient and average shortest path length of the observed network. Crand and Lrand are the corresponding averages from 500 Erdős–Rényi random networks with the same number of nodes and edges. The ‘Random Networks Used’ column indicates the number of successfully generated connected random networks (disconnected networks were excluded). The small-world coefficient is defined as σ=(C/Crand)/(L/Lrand). 2005 is omitted because the network was not fully connected at that time, making the average path length incalculable.
