# Supplementary figures and images for: From Centralized to Distributed Entropy: Long-Term Resilience and Structural Evolution of Regional Innovation Networks in the Yangtze River Delta
Source: Entropy (Basel). 2026 Jul 20;28(7):826. doi: 10.3390/e28070826 (PMC13409697; doi:10.3390/e28070826)

Figure S1. Yangtze River Delta Patent Cooperation Network (2024)

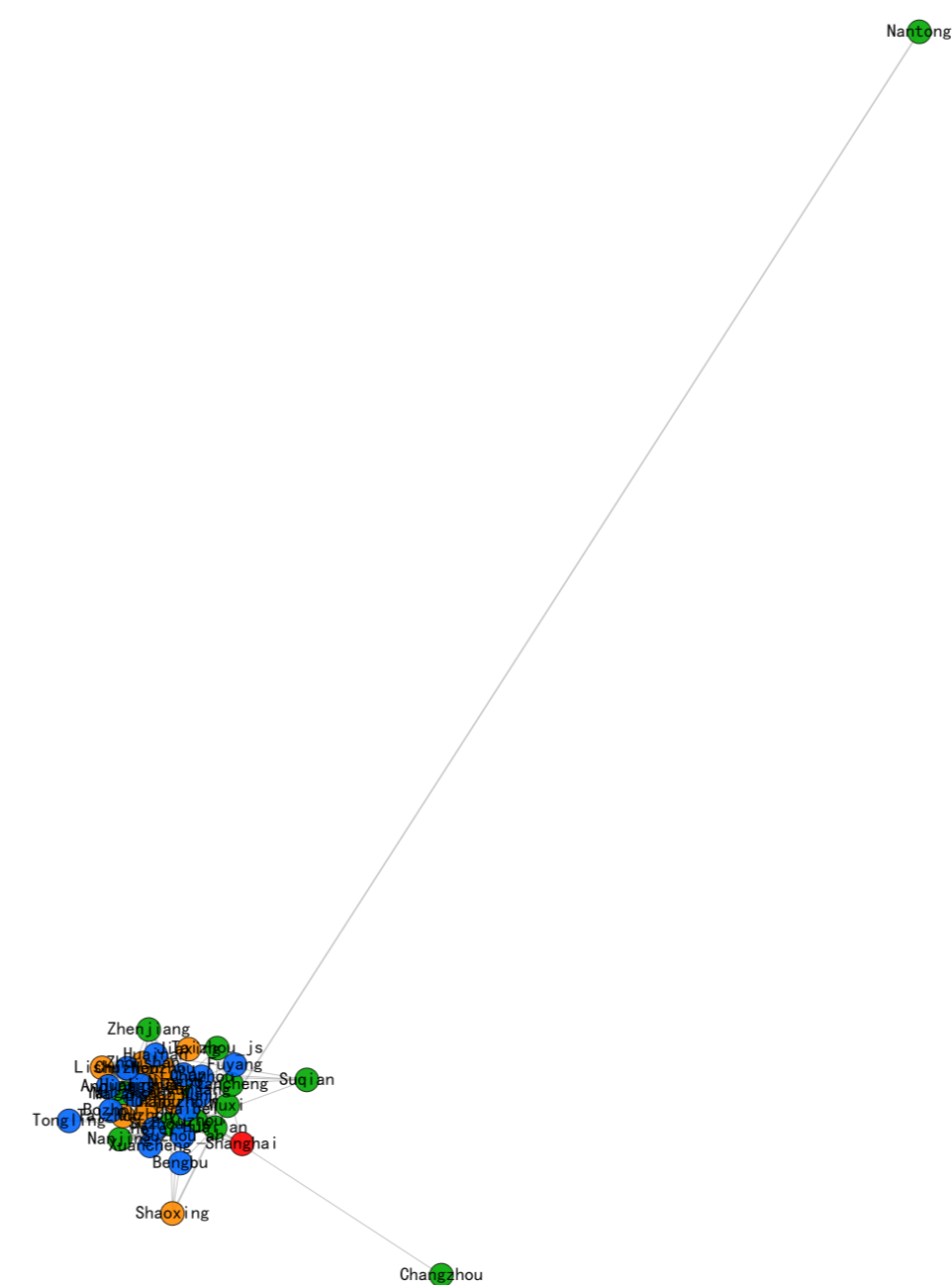

Supplement: Supplementary file 1 [file entropy-28-00826-s001.zip › supplementary material figure S1/Figure S1 Yangtze River Delta Patent Cooperation Network (2024).pdf]
